# Why media representations of corporations matter for public health policy: a scoping review

**DOI:** 10.1186/s12889-016-3594-8

**Published:** 2016-08-30

**Authors:** Heide Weishaar, Lori Dorfman, Nicholas Freudenberg, Benjamin Hawkins, Katherine Smith, Oliver Razum, Shona Hilton

**Affiliations:** 1 MRC/CSO Social and Public Health Sciences Unit, 200 Renfield Street, Glasgow, G2 3QB UK; 2 Berkeley Media Studies Group, Public Health Institute, and University of California, 2130 Center St. Ste. 302, Berkeley, CA 94704 USA; 3 City University of New York School of Public Health, 55 West 125th Street, New York, NY 10027 USA; 4 London School of Hygiene and Tropical Medicine, 15-17 Tavistock Place, London, WC1H 9SH UK; 5 2.27 Chrystal Macmillan Building 15a George Square, Edinburgh, EH8 9LD UK; 6 Bielefeld School of Public Health, Bielefeld University, Post box No.10 01 31, 33501 Bielefeld, Germany; 7 MRC/CSO Social and Public Health Sciences Unit, 200 Renfield Street, Glasgow, G2 3QB UK

**Keywords:** Media analysis, Corporations, Non-communicable diseases, Framing

## Abstract

**Background:**

Media representations play a crucial role in informing public and policy opinions about the causes of, and solutions to, ill-health. This paper reviews studies analysing media coverage of non-communicable disease (NCD) debates, focusing on how the industries marketing commodities that increase NCD risk are represented.

**Methods:**

A scoping review identified 61 studies providing information on media representations of NCD risks, NCD policies and tobacco, alcohol, processed food and soft drinks industries. The data were narratively synthesized to describe the sample, media depictions of industries, and corporate and public health attempts to frame the media debates.

**Results:**

The findings indicate that: (i) the limited research that has been undertaken is dominated by a focus on tobacco; (ii) comparative research across industries/risk-factors is particularly lacking; and (iii) coverage tends to be dominated by two contrasting frames and focuses either on individual responsibilities (‘market justice’ frames, often promoted by commercial stakeholders) or on the need for population-level interventions (‘social justice’ frames, frequently advanced by public health advocates).

**Conclusions:**

Establishing the underlying frameworks is crucial for the analysis of media representation of corporations, as they reflect the strategies that respective actors use to influence public health debates and decision making. The potential utility of media research lies in the insights that it can provide for public health policy advocates about successful framing of public health messages and strategies to counter frames that undermine public health goals. A better understanding of current media debates is of paramount importance to improving global health.

## Background

Research shows that the mass media, including print, radio, television, internet and social media, play a crucial role in framing public and political debates [1, 2]. They shape public perceptions by choosing which issues are reported as news and how these issues are represented, thus contributing to the definition and understanding of problems and their potential policy solutions [3–5]. Research also suggests that policy attention rises and falls in response to shifts in media coverage rather than with any change in the actual size of the problem [6], and that both policymakers’ perceptions of policy issues and the public’s acceptance of potential policy responses are considerably influenced by media debates [7–10].

One important aspect of media coverage relates to the way in which different kinds of stakeholders are portrayed since this influences their acceptability and legitimacy as societal and political players, and therefore, policymakers’ willingness to be associated with, or supportive of, them [11]. Correspondingly, regulation that penalises groups portrayed as worthy of punishment may be presented in positive terms and therefore find support with policymakers [12]. By shaping understandings and beliefs in relation to health issues, public policies and stakeholders involved in political decision-making, the media thus fulfill an important function in influencing the interactions between the public and political decision-makers. While the literature suggests that media debates should be a key concern for those interested in understanding or influencing public health policy processes, as yet there has been only limited research into the role of the media in the development of public opinion, advocacy, and policy in this area. This gap deprives those seeking to improve population health of relevant evidence to guide strategy.

In recent decades, non-communicable diseases (NCDs) have moved upwards in the ranking of causes of global years of life lost [13], becoming the world’s primary causes of death, disease, and disability [14]. Given that NCDs have been identified as one of the main global public health challenges of the 21st century, tackling their risk factors is a pressing political task [15]. In this context, it has been argued that more attention needs to be paid to tobacco, alcohol, processed food and soft drinks as modifiable NCD risk factors and to the industries that produce these products as key drivers of these epidemics [16–19].

While research examining the tobacco industry has led the way in helping the public and policy makers understand the detrimental impact of commercial interests on public health [20], more recently other industries, including alcohol, processed food and soft drinks corporations, are receiving increasing attention [21–24]. Of note is that corporate interests often run counter to public health interests [21, 25]. In order to maintain positive public and political perceptions of their activities, advance their business and political goals, and influence public and political debates, different industries employ similar political strategies and practices. As well as direct lobbying to undermine or prevent health regulation that is perceived to impact on profitability, common corporate tactics for achieving policy influence include ‘credibility engineering’ [26], emphasising ‘corporate social responsibility’ (CSR), and positioning corporations as credible societal and political partners in the development and implementation of public health policy [2, 18, 27–32]. A key corporate strategy closely linked to these tactics is framing public and political debates to align with commercial interests. Framing involves the generation of beliefs and ideas that provide a structure for thinking about an issue [33] and the construction of issues which direct attention towards particular aspects and ways of thinking about an issue (and away from others). Due to its power in forming particular perceptions about issues among target audiences [34] and shaping public views, voting patterns and political strategies, framing has been described as a key ‘weapon of advocacy’ and a potent tool to influence public and political debate [35, 36].

Given the pressing nature of the NCD crisis, the involvement of corporations in framing NCD-related problems, drivers and potential policy solutions, and the importance of the media in public health debates, media analyses can provide valuable insights into factors which crucially shape public and political debates on NCD risks and policies and the roles of different stakeholders in the development and implementation of such policies. Such analyses would also illuminate attempts by corporate stakeholders to influence and shape media coverage.

In this article, we review existing media studies on NCDs, focusing on how tobacco, alcohol, and processed food and soft drinks corporations (i.e. the industries marketing commodities that increase NCD risk) are portrayed in media coverage. We also examine how these corporate actors attempt to frame NCDs within this coverage, as compared to public health actors, and reflect on the underlying values that relate to different frames. The aim of this review is to identify gaps in the available research that seem useful to address, thereby setting the scene for future research which more fully examines media representations of industries that contribute to NCD risk and their impact, including how media representations might be shaping public and political opinion and, therefore, viable policy options.

## Methods

A scoping review was conducted to identify studies providing information on media representations of NCD risks, NCD policies and tobacco, alcohol, processed food and soft drinks industries. We searched Web of Science, Medline, Embase, and Google Scholar, using the following search terms in various combinations: tobacco, nicotine, alcohol, food, soft drink, beverage, nutrition, business, companies, company, industr*, corporate, corporation, commercial, communications media, mass media, media advocacy, document*, media, newspaper*, misinformation, policies, policy, policy maker*, policy actors, policy framing, public relations. All abstracts were read and studies were included in the final sample that met the following inclusion criteria: (i) published in a peer-review journal; (ii) applied media analysis as part of the methodology; (ii) provided information on: media representations of tobacco, alcohol, processed food or soft drink consumption; OR tobacco, alcohol or obesity policy; OR the tobacco, alcohol, processed food or soft drinks industry; (iv) published in English. No time or geographical limits were set. The bibliographies of included studies were checked for additional studies which met the inclusion criteria. The final sample was 61 studies, from which we systematically extracted the following data: primary product of interest of the study (tobacco, alcohol, processed food/soft drinks); specific issue investigated; location of study; period of investigation; type of media analysed; methodology; aim of the study; and findings on the media representation of tobacco, alcohol, processed food and soft drinks industry. We then synthesised the data narratively by developing a preliminary synthesis of the findings of each included study, exploring relationships, similarities and differences in the data, and summarizing the accounts of the depictions of the tobacco, alcohol, processed food and soft drinks industry in a way which accounted for the heterogeneity of studies. In a next step, a conceptual frame derived from the literature theorizing about framing of public health issues was applied to the data [37]. This conceptual frame which distinguished between social justice and market justice framing helped to categorise the two main opposing viewpoints identified in the media and make sense of the diversity of the data. To explore changes in the frequency of media coverage over time, a Poisson regression was used to examine the degree to which the frequency of articles was predicted by the year of publication.

## Results

### Description of the sample

Of the 61 studies, 40 analysed media coverage of tobacco, tobacco-related health problems, tobacco control policy or other tobacco-related issues; 12 analysed media coverage of processed food, soft drinks, obesity and other health issues related to unhealthy diet, or obesity and nutrition policy; and nine analysed media coverage on alcohol, alcohol-related health problems or alcohol policy. None of the studies explicitly compared NCD risks, policies, or corporations/industries across sectors. An inductive analysis of the data that were extracted from each article showed that media analyses primarily focused on one of the following topics: harms and health conditions caused by tobacco, alcohol, processed food and soft drinks (e.g. second-hand smoke, tobacco-related disease, binge drinking, obesity), public health policies (e.g. smoke-free bars, soda tax, minimum unit pricing), industries (e.g. tobacco companies, litigation against tobacco companies), consumers (e.g. smokers, drinkers), and products (e.g. tobacco, alcohol, processed food, soft drinks). Most (*n* = 49) studies focused on the harms and health conditions caused by, and the regulation of, tobacco or alcohol or processed food and/or soft drinks consumption. Only six articles explicitly analysed the representations of corporations in the media (*n* = 2) or media coverage of litigation against corporations (*n* = 4), and all of these focused on tobacco companies (Table 1).

**Table 1:** Main foci of media analyses of NCDs, 1981-2015

Main focus of media analysis	Number of studies
Harms and health conditions caused by products	26
Public health policies	23
Corporations	6
Consumers	3
Products	3
Total	61

**Fig. 1:**
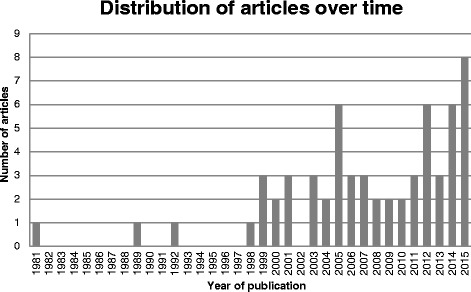
Distribution of media analyses of NCDs over time, 1981–2015

The earliest media analysis of NCD risk in our sample – reporting on a study assessing coverage of tobacco hazards in women’s magazines – was published in 1981. Only two articles were published in the 1980s and five in the 1990s, but publication frequency increased from 1998 onwards to a peak of eight in 2015. Between 1981 and 2015 there was a highly significant upwards trend in the number of studies analysing NCD media debates published per year (Poisson regression coefficient 0.113, *p* < 0.000) (Fig. 1). All articles but one that were published prior to 2005 focused on tobacco-related issues, suggesting that research interest in media representations of NCD risks initially concentrated on tobacco and only recently broadened to alcohol, processed food and soft drinks.

In terms of geographical focus, studies overwhelmingly analysed media in English-speaking countries, including the USA (*n* = 39), Australia (*n* = 12) and the UK (*n* = 7), possibly reflecting the fact that searches were undertaken in English. The remaining four articles focused on media coverage in Finland, Switzerland, Hong Kong and internationally. Only one study compared media across countries, focusing on US and Australian media coverage of tobacco.

More than half of the articles (*n* = 35) considered newspapers only. Other studies combined newspaper analysis with one or two other data sources, including TV programmes (*n* = 10), magazines (*n* = 8), trade or industry documents, websites or journals (*n* = 5), policy documents (*n* = 5), radio (*n* = 2), press releases (*n* = 1) or interview data (*n* = 1). Two studies investigated magazine articles only, and one article was unclear about the specific media sources that were included in the analysis. We found a striking dearth of studies taking account of online and social media. Only three studies which assessed multiple data sources compared reporting across, and associations between, different data sources, e.g. between newspaper coverage and industry documents, policy documents or personal accounts [38–40].

Seven studies investigated media coverage over a time period of less than a year (with *n* = 3 articles investigating media coverage during one month only); 30 studies investigated media coverage over a time period of 1 to 5 years; 11 studies investigated media coverage over a time period of 5 to 10 years; and 13 studies investigated media coverage over 10 years or more. In some instances, choosing a specific period of time was rationalised by the occurrence of specific events that were of interest to the subject of study, e.g. the release of a policy report or the adoption of legislation. More frequently, however, choices seemed to be for pragmatic reasons.

Methodologies used to analyse media coverage ranged along a continuum from more narrowly deductive analyses to very open, inductive analyses. Researchers, for example, employed quantitative analysis, semantic analysis, content analysis based on stringent coding frameworks, ethnographic content analysis, and discourse analysis as well as a combination of these methods. While all of these approaches are potentially appropriate, a lack of acknowledgement of alternative analytical approaches in individual studies, or of cross-referencing between these different methodological approaches, suggests that this is an area of research which would benefit from greater methodological cross-fertilization.

### Media depictions of industries

The amount and nature of information on the representations of tobacco, alcohol, processed food and soft drinks industries in media debates differed widely among the 61 studies. Strikingly, 13 studies did not mention corporate stakeholders at all. The 48 studies that considered the representation of industries tended to approach the subject in one of two ways: 38 studies described corporate attempts to frame debates on tobacco, alcohol, processed food and soft drink consumption and related policies; ten studies explicitly assessed the media’s representations of industries themselves, e.g. reflecting on whether industries were portrayed positively or negatively or on whether attention was drawn to corporate conduct and the industry’s role in NCD debates. Of these ten studies, seven focused on the tobacco industry and three focused on the representations of processed food or soft drinks industries in the media. While the data suggested that tobacco companies were largely portrayed in a negative light, with media drawing attention to corporate misconduct and the industry’s adverse impact on public health, soft drinks and processed food companies seemed to be portrayed less critically, with the media drawing less attention to their role in enhancing NCD risk. Several analyses of media debates on tobacco issues, for example, showed that media employed value-laden discourse to portray tobacco companies as causal factors of tobacco-related health problems and unscrupulous contributors to death and disease [11, 41–43]. On the other hand, a US media analysis highlighted that media discourse focused primarily on individual-level causes of obesity and only shifted to a focus on systemic causes after the launch of a campaign for a reduction in sugar-sweetened beverages when news stories began to be “significantly more likely to mention food and beverages companies as contextual agents of obesity” [44].

The conceptual frame from previous literature which distinguished between market justice and social justice helped to further analyse media discourse on NCD risks and policies. The analysis showed that while corporations seemed to focus largely on individual responsibilities and draw on market justice frames when talking about NCDs, public health advocates seemed more likely to promote population-level interventions and structural changes and frame debates around social justice issues. Table 2 categorises the findings from the scoping review into these two dominant frames, outlines the arguments put forward by the proponents of the two frames divided by product/policy area, and identifies common rationales across product/policy areas which underlie each frame. The following section elaborates on each of the two framing strategies in more detail.

**Table 2:** Overview of ‘Market Justice’ and ‘Social Justice’ Framing of Tobacco, Processed Food and Soft Drinks, and Alcohol issues adapted from: [18, 34, 37, 82]

	Tobacco	Processed food and soft drinks	Alcohol	Common
Market Justice	• Tobacco consumption is a choice; p eople chose to smoke. • People have a right to smoke. • Parents are responsible for ensuring that their children don’t smoke. • Tobacco companies provide a product which people chose to consume. • Tobacco companies have a right to promote a legal product. • Sufficient legislation is in place to prevent children and adolescents from accessing tobacco; additional legislation is unnecessary. • Tobacco companies, particularly those that produce products with less harm than traditional cigarettes, are legitimate partners in tobacco control policy and harm reduction strategies.	• People should be able to choose what they eat freely. • Parents are responsible for ensuring that their children eat healthy diets. • What the food industry produces reflects what people want. • Processed food and soft drinks companies are legitimate and crucial partners in the development and implementation of obesity and nutrition policies.	• Individuals are responsible for drinking alcohol safely and responsibly. • Parents are responsible for ensuring that their children don’t drink alcohol. • The alcohol industry is not responsible for the irresponsible or dangerous behaviour of ‘problem drinkers’ (e.g. binge drinkers). • Sufficient legislation is in place to prevent children and adolescents from accessing alcohol; additional legislation is inappropriate. • Alcohol companies are legitimate and crucial partners in the development and implementation of alcohol policies.	• Individuals are responsible for their own well-being. • The best solutions to public health problems are individual level approaches. • Tobacco, alcohol and food and drinks consumption are lifestyle issues. • Resources should be allocated based on ability to pay, not need. • Market forces are a suitable means to determine that the right products are available to the appropriate customers. • Industry can help people make informed personal choices by providing information. • Voluntary codes and self-regulation are more efficient, effective and appropriate than government regulation. • ‘Nanny state governments’, that regulate what individuals can and cannot consume, deprive people of their freedom and liberty and coddle people, preventing them from the dignity of fending for themselves.
Social Justice	• People have a right to breathe air uncontaminated by second hand smoke. • Communities have a right to say no to targeted marketing of tobacco. • Vulnerable populations, e.g. children and adolescents, have to be protected from marketing of tobacco, whose aim is to initiate and maintain addiction. • Regulation of the industry is crucial to curtailing the epidemic. • The tobacco industry has a responsibility to pay the costs of tobacco-related illnesses. • Tobacco policy has to be protected from the vested interests of tobacco companies.	• All people and communities have a right to have access to healthy and affordable food. • Food companies should not promote unhealthy products, particularly not to children. • Vulnerable populations, e.g. children and adolescents, have to be protected from targeted marketing of unhealthy food and drinks. • Regulation of the industry is necessary to curtail obesity. • Due to their commercial interests, processed food and soft drink industries should not be able to influence obesity and nutrition policies.	• Communities and governments have a duty to protect citizens from risky alcohol use. • Alcohol companies should not promote their products to children, adolescents, or problem drinkers. • Vulnerable populations, e.g. children and adolescents, have to be protected from alcohol marketing, which aims to encourage unhealthy alcohol consumption and initiate and maintain addiction. • Regulation of the industry is necessary to curtail harmful consumption of alcohol. • Due to their commercial interests, alcohol companies should not be able to influence alcohol policy.	• Members of society have a shared responsibility to look after each other. • Vulnerable populations have to be protected from exploitation by more powerful societal actors. • Public policy should make healthy behaviour the easier and more accessible choice. • When markets fail to protect public health, communities and governments have a right and responsibility to act, e.g. by regulating industries and preventing corporations from influencing public health policies.

### Corporate attempts to frame debates

Arguably one of the most striking finding was that the views that were voiced by corporate representatives about tobacco, alcohol, processed food or soft drink consumption and related policies were almost uniform across these industries. In almost every case, corporate stakeholders made arguments in the press that emphasised the logic, value and inherent morality of the unfettered marketplace and countered population-based public health policy proposals by focusing attention on unhealthy lifestyles, individual responsibility, consumer choice and economic arguments. In media studies that described how stakeholders tried to frame debates, corporate stakeholders were reported to characterise consumption primarily as a personal choice, an individual level responsibility and a moral issue. If consumption was accepted as problematic, corporate representatives described it as the behavioural problem of an individual, rather than a societal problem. Corresponding to such discourse, commercial representatives uniformly opposed population-level tobacco, alcohol or obesity policies, like smoke-free legislation, minimum unit pricing for alcohol (MUP), or soda tax. In order to divert attention away from corporate actions as potential causes and oppose government regulation, corporate stakeholders presented arguments that such policies would have detrimental effects on the economy, were disproportionate, unnecessary, not feasible, or an example of ‘nanny state’ regulation and intrusion into personal freedom. They also promoted voluntary regulation as an alternative to legislation. The two studies which investigated industry reporting across different types of media found that the financial and trade press and media outlets which relied heavily on advertising income were more likely to present such arguments [45, 46].

Examples of corporate framing strategies of NCD policy debates were identified in studies which analysed the alcohol industry’s use of the media. Katikireddi et al.’s [47] work analysing recent UK policy debates on MUP, for example, showed that industry representatives were instrumental in publicly defining the problem of excessive alcohol use as largely being restricted to ‘binge drinkers’ and ‘dependent drinkers’ rather than a widespread population level problem. In efforts to oppose public health frames, which defined alcohol consumption as a population-level problem needing a population level intervention (such as MUP), alcohol industry stakeholders argued for targeted approaches to educate individuals [47]. Hawkins and Holden’s [48] analysis of UK debates on MUP policy similarly identified corporations’ use of the media to promote their specific framing of alcohol-related harm as a matter of individual responsibility and highlight the importance of the alcohol industry for the UK economy and the alleged negative side-effects of over-regulation. Arguing that harms were restricted to a small minority of harmful drinkers who should be the focus of targeted policy interventions, industry stakeholders used CSR to buttress calls for industry self-regulation and public information/education as direct alternatives to MUP [48–50]. Yoon and Lam [51] identified similar efforts by the alcohol industry to shift the blame to the consumer and promote voluntary regulation across the globe via trans-national industry bodies. Mirroring framing strategies of the alcohol industry, two US studies investigating the introduction of soda taxes as a measure to reduce obesity showed that soda industry spokespeople framed their comments to focus debates on individuals’ personal responsibilities for their health behaviour. They also showed that arguments were presented on the need to protect citizens from government overreach and of companies being good corporate citizens who were ‘part of the solution’ to reducing obesity [52, 53].

### Public health attempts to frame debates

In contrast to the industry framing strategies on NCD-related debates, our review identified a similarly consistent alternative framing strategy most often promoted by public health advocates. Advocates used the media to advance arguments to emphasise the social and political determinants of health, the detrimental impact of industries producing and marketing tobacco, alcohol, processed food and soft drinks on NCD-related harm, and the need for regulation and population-based interventions. A number of studies, most prominently those that analysed tobacco debates, showed that some media depictions emphasised the tobacco industry’s mendacious conduct, corporate representatives’ ruthless marketing of a lethal product to children and vulnerable populations, and their focus on profits and economic interests ahead of any moral considerations [54, 55]. Supporting such media discourse, another study identified the representations of the smoker as a tobacco industry pawn as a media frame which drew attention away from individual responsibility and towards the misconduct of the industry [41]. An article by Christofides and colleagues [11], which investigated the tone in which a leading Australian newspaper portrayed the tobacco industry between 1993 and 1997, revealed primarily negative depictions of tobacco corporations.

Several media studies showed that as well as drawing attention to industries as causal factors of tobacco, alcohol and nutrition-related health problems, public health advocates often called for structural changes or the regulation of the industry and its products. For example, Lawrence [56] examined frames of obesity in front page stories and editorials in the *New York Times* from 1985 to 2003, and described public health advocates’ attempts to advance “arguments emphasizing the social environment, including corporate and public policy”, tailored to denouncing corporate practices and calling for regulation of soft drinks. Similarly, two studies, one on media coverage of New York sugar-sweetened beverage portion-size cap policy [57] and another on a sugar-sweetened beverage reduction campaign in Philadelphia [44], mentioned that the industry was presented in some media reports as a contextual agent and industry supersizing as a driver of the obesity epidemic. However overall, media analyses of alcohol, processed food and soft drink debates seemed to report less frequently on framing that exposed industry misconduct and its detrimental impact than did analyses focused on tobacco.

## Discussion

This scoping review indicates that the framing of media debates on NCD risk and policy is an under-developed area of research, and that we know particularly little about similarities and differences in the media strategies and portrayals of industries marketing commodities that increase NCD risk and how media coverage of these industries influences public and policy debates on NCDs. By undertaking a narrative review of a variety of studies, this paper enables us to make some comparisons and helps explain why the growing sense of caution regarding tobacco industry engagement with policy debates has not been expanded to other industries. Based on the review, we identify a need for a range of approaches to discourse analysis to support future research and practice on media coverage of industries involved in NCD debates. Our findings corroborate the call made by Australian academics more than two decades ago to take the framing of public health issues far more seriously [58], including via discourse analysis [59]. While a recent increase in publications suggests that awareness of the importance of the topic is growing among academics, 61 studies over the last 30 years seems inadequate. The limited body of literature highlights the need for more research about media representations and their role in public and political debates, especially as it is dominated by US based studies and studies focusing on tobacco. Our review suggests at least three aspects of research for developing an agenda to influence media debates on NCD risks and policy: (i) How are media debates framed and which stakeholders and other factors influence such framing? (ii) What impact does such framing have on the views, opinions and behaviors of the public and policymakers, including decision-making regarding NCD risks and potential solutions? (iii) How best can media analyses be used to investigate debates on NCD risks, policies, or industries across sectors?

Our review found that media depictions of NCD risks and potential solutions to them seem to reflect broader societal tensions between two different viewpoints. Opponents of public health policies tend to promote frames which place the focus on unhealthy lifestyles as individual level problems and portray individuals as making more or less informed choices to smoke, consume alcohol or unhealthy food and drinks. Proponents of these frames tend to support voluntary policies and interventions targeted at changing individual behaviour (e.g. education campaigns), rather than regulatory and systemic changes (since these tend to be viewed as anti-liberal). When this kind of framing dominates political and public debates about NCDs, corporations (often the most vocal supporters of these frames) tend to be portrayed as responsible, legitimate societal and political actors. Advocates of public health policies, on the other hand, often attempt to frame debates very differently, drawing attention to systemic, rather than individual, causes. They portray corporations as powerful, profit-driven actors who relentlessly market unhealthy products that undermine public health, and call on governments to assume responsibility to advance public health and protect citizens from dominant market interests by adopting regulation.

These contrasting frames identified with regard to media depictions of NCDs resonate strongly with work by Beauchamp [37], who identifies two opposing forces in society that impinge on public health, what he called “market justice” and “social justice”. He maintains that “[p]ublic health should be a way of doing justice [to assert] the value and priority of all human life” and argues that market justice is a direct challenge to this ethic in “unfairly protect[ing] majorities and powerful interests from their fair share of the burdens of prevention” [37]. Beauchamp’s depiction of market justice is rooted in the basic notion of Adam Smith’s [60] ‘invisible hand’, the idea that the market will naturally respond to the desires of the people and that the unfettered marketplace is the best way to serve those desires. Our review suggests that the degree to which one of the two frames dominates media coverage of NCD risk and solutions depends on the topic and context. Future research could explore which topic- and context-specific factors facilitate the dominance of one or the other frame.

The political implications of such different ways of framing are evident: Policymakers who are persuaded by ‘market justice’ framing are less likely to adopt policies that regulate corporate behaviour, whereas policymakers who follow ‘social justice’ framing will be more willing to restrict corporate abilities to market and sell products which contribute to the burden of NCDs cf. [34]. Drawing on the literature on framing of public and political debates on tobacco as an example, research highlights that, historically, tobacco corporations’ successful framing in terms of ‘market justice’ ideals, stressing personal freedom, economic growth, trade and CSR, has resulted in positive public and political perceptions of the industry and effective tobacco control legislation being delayed, withdrawn and amended [61–63]. However more recently, studies demonstrate that public health advocates in some (high income) contexts have been successful in framing at least some tobacco-related debates by focusing discussions on the right to breathe clean air, the need to protect vulnerable populations, and the effectiveness of comprehensive tobacco control, thereby advancing the adoption of comprehensive policies [64–66]. Most prominently, the success of advocates in shifting the frame from the industry supported value of the ‘right to smoke’ to the social value of the ‘right to breathe clean air’ played a key role in winning public support for stronger laws to reduce exposure to second-hand smoke [67]. Our review provides some indication that tobacco control media coverage has shifted towards a stronger emphasis on systemic factors. However, whether this is consistent across countries and contexts will have to be explored in more detailed analyses.

Recent research on framing contests in alcohol and obesity policies provide emerging evidence of similar framing challenges: Public health advocates’ recent framing of UK alcohol pricing and promotion policy as effective means to tackle population-level harms were important in the passage of evidence-based policy measures [48]. However, in some of the first soda tax debates in the US, advocates health arguments which supported excise tax measures aimed at curbing consumption of sugary beverages dominated news coverage, but ultimately, industry arguments prevailed with voters, and the taxes were defeated [52]. Learning from these prior efforts, public health advocates in Berkeley, California, adapted their organising strategies, mobilised more diverse and representative local spokespeople, focused attention on the untrustworthiness of the sugary beverage industry, dominated the debate in news coverage and on social media, and eventually won the first tax on sugary beverages in the US [68].

Beyond the finding that media coverage of NCDs and industries contributing to NCD risk occur within the tension of two rival frames, our review highlights considerable research gaps with regard to the values which underlie different frames; the contestations in media debates between industry and public health interests; the complex relationship between media coverage, public and political opinion, and policy debates and decisions; and the way in which changes to the media landscape may be complicating these interdependencies. Future studies could usefully investigate the kinds of questions outlined in Table 3.

**Table 3:** Research questions to investigate

• How are industries that contribute to NCD risk portrayed in the news media and how and why does this vary by sector and context (e.g. country or type of media outlet)? • What kind of social, political and ideological values (e.g. neoliberalism, health, justice, etc.) underpin varying media representations of industries and their roles in NCDs and policy responses to NCDs? • Do the business models and funding sources of media outlets affect the way in which corporate behaviour and responsibility for NCDs are framed? • How are public health interests, corporate actors and government each portrayed in the media in relation to NCD policy debates? • To what degree do variations in media coverage of industries involved in NCD debates interact with changes in public opinion, policy opinion and broader societal values? Is it possible to identify pathways and directions of influence? • Which factors contribute to the success (or otherwise) of strategies employed by corporate, government and public health interests to influence media debates about NCDs? Do they vary by sector? • What evidence is there of shared learning/resources across industries in media debates about NCDs? • How do challenges to traditional media (e.g. ‘citizen journalism’, ‘advertorials’, native advertising, organizational subscriptions and the rise of social media) change the media representations of industries that contribute to NCD risk? • How can those who try to counter corporate framing of policy debates and corporate influence on public health use traditional and social media to reframe these debates?	

Our review identifies a particularly urgent need to address the striking gap in terms of comparative research on the representations of different industries whose products increase NCD risk. The considerable variations in perceptions that public and political audiences seem to have of the tobacco industry compared to alcohol, processed food and soft drinks corporations [7] underlines the need for comparative research which aims to better understand the basis on which distinctions among these different industries are being made in popular media. Our scoping review suggests that media debates on alcohol- and nutrition-related harms less frequently focus on the detrimental effects of commercial interests on public health and that media representations of alcohol, processed food and soft drinks companies overall seem to be predominantly positive. While it is not possible, without further research, to ascertain the extent to which these contrasting representations shape the decisions of policymakers (or are shaped by those decisions), it is certainly the case that alcohol and food companies are currently treated very differently from tobacco companies in many policy contexts, with alcohol and food companies being perceived as legitimate stakeholders and policymaking partners in NCD debates in the UK [69], US [70] and internationally [15]. Given that previous research suggests that tobacco advocates’ increasing confidence to speak out about the industry’s detrimental influence on public health has been crucial in increasing public and political awareness and support for population-based tobacco control measures [66, 71], comparative research on media representations of tobacco, alcohol, processed food and soft drinks industries is likely to offer opportunities for other areas of public health to learn from tobacco control strategies.

This scoping review also provides direction in terms of potential methodological approaches. Media studies scholars have employed a wide variety of approaches to analysing text and speech, including Critical Discourse Analysis [72, 73] and semiotic discourse analysis [74]. Although the 61 studies we identified for this review were dominated by qualitative analyses of media debates, the depth of most analyses was limited (often as a consequence of the amount of data), suggesting that there is scope for future media analysis studies to make better use of more in-depth discourse analysis techniques. In-depth discourse analysis could, for example, employ comparative approaches and combine an analysis of the structure and content of media texts with an analysis of the wider context in which these texts are produced, including the media context (e.g. investigating potential correlations between framing and the media outlet’s business models and funding sources) and the political context (e.g. examining political and ideological values and the adoption and enforcement of NCD policies). Such qualitative approaches are usefully complemented by quantitative analysis of media discourses to provide an overview of changes in coverage over time and the relative prominence of different stakeholders and their arguments in media debates within the broader policy landscape [5, 75]. Statistical techniques, like the regression analyses conducted as part of this paper, can be useful for analysing trends in topics over time, indicating trends away from a focus on individual level policies towards greater levels of reporting on societal solutions such as regulatory change (as found in the news analysis of media discourse around a need to regulate obesogenic environments [5]) and mapping changes in media attention to policy debates and advocacy.

Existing media studies further show that researchers need to carefully tailor the selection of media outlets and outputs to the audiences that are the focus of investigation. Scholars who are interested in the role of media representations in policy debates should, for example, identify and select media outlets and outputs that are read, viewed, and listened to by the public officials, politicians and advocates in whom they are interested. This is likely to include some traditional print media sources, for which methodological approaches have been well developed [76–78]. However, considering the rapidly changing online media landscape and lack of studies which analyse corporate representations using social media and online media, it is likely that methodological approaches need to be developed further to capture these diverse and dynamic platforms. With declining revenue for traditional news media, the rise of social media, and a move to ’24 h’ broadcast and internet news, journalists are under pressure to produce more material faster, and with fewer resources. This means journalists often have less time to research issues in any depth and increasingly rely on externally produced stories and information [79]. Simultaneously, research shows that industry representatives are very aware of the media’s importance in shaping public opinion and use it tactically to influence public and political debates [80]. Therefore, highly profitable tobacco, alcohol, processed food and soft drink corporations can exploit increasing opportunities to supply journalists with material on which to base articles. Lewis et al.’s [81] comparative analysis of articles appearing in British daily newspapers which identified the extent to which media outlets rely on external material, including press releases and corporate public relations materials, showed that in many instances, media texts could be traced back verbatim to industry press releases.

Studies which analyse media coverage over time and relate findings to corporate or political discourses and outcomes by combining analyses of media coverage, press releases, policy documents and interviews are essential if we are to make any progress in identifying the influence of corporations on media representations or the contribution of media discourses to evolving policy debates. Such studies can illuminate the complex relationship among media coverage, public and political opinion, and political decision making. The broader social sciences provide an array of potentially useful additional methodological approaches, including innovative use of plagiarism detection software to analyse similarities between political statements [82], an approach which could easily be developed to analyse similarities between media stories and the press releases of corporations or policy documents.

## Conclusions

In conclusion, studies that investigate media debates on NCD risk and policy are important for developing a more nuanced understanding of the complex ways in which media representations of unhealthy commodity industries are shaped by, and contribute to shaping, public, corporate and political discourse. We urge those who are interested in this area of scholarship to consider the potential utility of media research for public health policy advocates. Media studies, including those about how highly skilled corporate stakeholders or successful public health advocates articulate their political messages, can provide insights into how to frame effective public health messages and counter frames that undermine public health goals. A better understanding of current media debates can improve the development and dissemination of framing NCD-related issues and is of paramount importance to improving global health. We have provided an overview of what is currently known about framing of NCD risks and policies in the media and media representations of industries that contribute to NCD risks, identified a set of important questions to consider in future media analyses looking at NCDs, and outlined implications for public health policy and advocacy. Our findings and reflections could support future research in this area, provide a crucial resource for those seeking to develop a common policy agenda to reduce NCD-related harm, and enhance public health advocates’ abilities to use the media to promote effective public health policy.
